# Comparative Evaluation of Cone-Beam Computed Tomography (CBCT) and Panoramic Radiography for Presurgical Implant Planning in the Posterior Mandible: A Retrospective Analysis

**DOI:** 10.7759/cureus.117151

**Published:** 2026-09-29

**Authors:** Sowmiya Elango, Varun Muthuraman, Sri Sahana Ilango, Venkateshpraveen M, Vijay Thiyagarajan J, Rashmi Priyem Saravanan Vellaiappan

**Affiliations:** 1 Oral and Maxillofacial Surgery, Best Dental Science College and Hospital, Madurai, IND; 2 Oral and Maxillofacial Surgery, JKKN Dental College and Hospital, Komarapalayam, IND

**Keywords:** bone density, cbct, implant planning, panoramic radiography, posterior mandible

## Abstract

Background: Radiographic evaluation is critical in implant planning in the posterior mandible, where proximity to the inferior alveolar neurovascular bundle necessitates precise assessment of bone quantity, bone quality, and spatial relationships with vital anatomical structures. Panoramic radiography is widely available and cost-effective but suffers from magnification distortion and inability to depict buccolingual dimensions. Cone-beam computed tomography (CBCT) provides three-dimensional (3D) information but at a higher cost and radiation exposure.

Objective: This study aims to compare CBCT and panoramic radiography in terms of (i) visualization of the mandibular canal and mental foramen, (ii) visualization of the crest of the alveolar ridge and superior border of the inferior alveolar canal, and (iii) assessment of bone quantity (height and mesiodistal width) and quality (buccolingual width and density in Hounsfield units (HU)) at posterior mandibular implant sites.

Methods: A retrospective cross-sectional analysis was performed on pre-existing radiographic records from a single academic center, comprising CBCT volumes and corresponding panoramic radiographs of the same patients with at least one posterior mandibular edentulous site. Visibility of the mandibular canal at the mental foramen and at 1, 2, and 3 cm posterior to the mental foramen was graded using a four-point scale (1, poor; 4, excellent). Visibility of the superior border of the inferior alveolar canal and crest of the alveolar ridge was similarly graded. Bone height and mesiodistal width were measured on both modalities, whereas buccolingual width and bone density (HU) were obtained only from CBCT.

Results: CBCT showed significantly higher grading scores for mandibular canal visualization at all four evaluated locations (at the mental foramen and at 1, 2, and 3 cm posterior) compared with panoramic radiography. Visualization of both the crest of the alveolar ridge and the superior border of the inferior alveolar canal was also significantly better with CBCT.

Conclusion: CBCT provides superior visualization of the mandibular canal in the posterior mandible, especially 2-3 cm distal to the mental foramen, and uniquely allows assessment of buccolingual bone dimensions and density, making it more suitable than panoramic radiography for presurgical implant planning in anatomically critical posterior mandibular sites. Panoramic radiography remains acceptable for routine cases with adequate vertical bone height away from vital structures, but CBCT should be considered when canal visualization is inadequate or when buccolingual and density information are required.

## Introduction

Branemark and colleagues' pioneering work on osseointegration established the biological basis for endosseous implant therapy and ushered in the modern era of dental implantology. Their histologic observations of direct contact between living bone and the implant surface have translated into predictable long-term outcomes for rehabilitation of partially and completely edentulous patients. Compared with conventional prosthodontic options, implant-supported restorations often provide more stable and durable functional and aesthetic results [1].

However, implant placement in the posterior maxilla and mandible presents particular challenges. In the posterior maxilla, relatively poor bone quality and reduced bone volume due to maxillary sinus expansion and alveolar ridge resorption contribute to lower long-term success rates compared with other regions [2]. In the posterior mandible, the close relationship of potential implant sites to the mandibular canal and mental foramen demands careful preoperative localization of the neurovascular bundle to avoid sensory disturbances.

Successful implant therapy depends on accurate assessment of the available bone support. Effective presurgical planning requires information about bone height, width, contour, density, and the position of anatomical landmarks, alongside clinical factors such as number of implant sites, need for augmentation, and consideration of radiation dose and cost when selecting the imaging modalities [3]. Radiographs are integral to all phases of implant treatment-initial evaluation, surgical placement, and long-term maintenance.

Conventional two-dimensional (2D) techniques, including periapical, bitewing, occlusal, cephalometric, and panoramic radiography (OPG), provide valuable information yet are inherently limited. OPGs offer a broad "bird's-eye" view of the jaws with relatively low radiation but cannot depict buccolingual bone dimensions and are subject to magnification and distortion, especially when patient positioning is suboptimal [4]. Advanced imaging techniques such as conventional tomography (CT), multislice computed tomography, magnetic resonance imaging (MRI), and cone-beam computed tomography (CBCT) overcome some of these limitations [5].

CBCT has gained prominence in implant dentistry because it provides cross-sectional and volumetric views of the dental arches, allows visualization of alveolar process inclination, facilitates precise measurements of bone quality and quantity, and improves localization of the mandibular canal [6]. Modern CBCT systems use flat-panel detectors and image processing algorithms that yield high-resolution images with isotropic voxel size and reduced geometric distortion. At the same time, concerns persist regarding radiation exposure cost and the need to balance diagnostic benefit against the "as low as reasonably achievable (ALARA)" principle.

Against this background, the present study was designed to compare the efficacy of OPG and CBCT for implant planning in the edentulous or partially edentulous posterior mandible, focusing on visualization of vital structures and quantitative assessment of local bone dimensions and density.

## Materials and methods

Study design and setting

This retrospective cross-sectional study was conducted in the Department of Oral and Maxillofacial Surgery in collaboration with the Department of Oral Medicine and Radiology at Sree Mookambika Institute of Dental Sciences, Kulasekharam, Tamil Nadu, India. Archival radiographic records obtained for diagnostic purposes and stored in the institutional database were reviewed. This study aimed to compare vertical height and mesiodistal width between modalities and evaluate the unique diagnostic yield of CBCT for buccolingual width and bone density

Sample selection

Radiographic records of patients aged 18 years and above with at least one edentulous area in the posterior mandible suitable for hypothetical implant placement were screened. For inclusion, each case required both the CBCT and a conventional OPG of adequate diagnostic quality covering the region of interest. Patients with developmental anomalies, maxillofacial bony lesions, fractures, prosthetic jaws, or severe systemic conditions that could interfere with imaging were excluded. After applying inclusion and exclusion criteria, a final sample of posterior mandibular edentulous sites with paired CBCT and panoramic images was selected for analysis. This was a paired, within-subject design. Each of the 26 patients underwent both CBCT and OPG of the same posterior mandibular edentulous site(s), yielding 52 paired observations for comparative analysis.

Sample size calculation

A pilot study was conducted on five patients (not included in the final analysis) to estimate the parameters for sample size calculation. The mean canal visibility score at 2 cm posterior to the mental foramen was 3.2 ± 0.8 for CBCT and 2.1 ± 0.9 for panoramic radiography. The standard deviation of the paired differences was calculated as 0.665, assuming a correlation coefficient of 0.7 between paired measurements. Using a two-tailed paired-difference formula with α = 0.05, Zα/2 = 1.96 (95% confidence), β = 0.20, Zβ = 0.84 (80% power), and a prespecified clinically meaningful difference of 0.37 units: n = [(Zα/2 + Zβ)² × σd²]/d² = [(1.96 + 0.84)² × 0.665²]/0.37² ≈ 25.3, rounded up to 26. The final study enrolled 26 patients, yielding 52 paired observations (26 with OPG and 26 with CBCT of the same posterior mandibular sites).

Imaging protocols

OPGs were acquired using a Planmeca Proline XC unit on green‑sensitive Kodak T‑MAT films (15 × 30 cm) following manufacturer‑recommended exposure parameters and standard patient positioning instructions. CBCT scans were obtained using a Planmeca ProMax® 3D Max machine, with the field of view adjusted to include the posterior mandible and exposure parameters optimized for diagnostic quality while respecting radiation safety principles. Digital Imaging and Communications in Medicine (DICOM) data from CBCT scans were reconstructed and analyzed using Planmeca Romexis Viewer software (version 5.30.1015).

Qualitative Assessment of Mandibular Canal Visibility

Both panoramic radiographs and CBCT images were evaluated for visibility of the mandibular canal at four standardized locations: at the mental foramen and at 1, 2, and 3 cm posterior to the mental foramen along the body of the mandible. Visibility was graded using a four-point ordinal scale adapted from the methods described by Kamrun et al. and Jung and Cho [7-8].

Poor: canal is not discernible, and the outline is indistinguishable from surrounding structures.

Severe limitation: localization is not possible; discontinuity >3 mm.

Slightly limited: artifacts or short discontinuities of 1-3 mm or mild contour distortion.

Excellent: continuous, smooth, and well‑defined canal outline.

This grading scale was applied independently on panoramic photographs and CBCT cross-sectional or reconstructed panoramic views (Figures 1-2).

**Figure 1:**
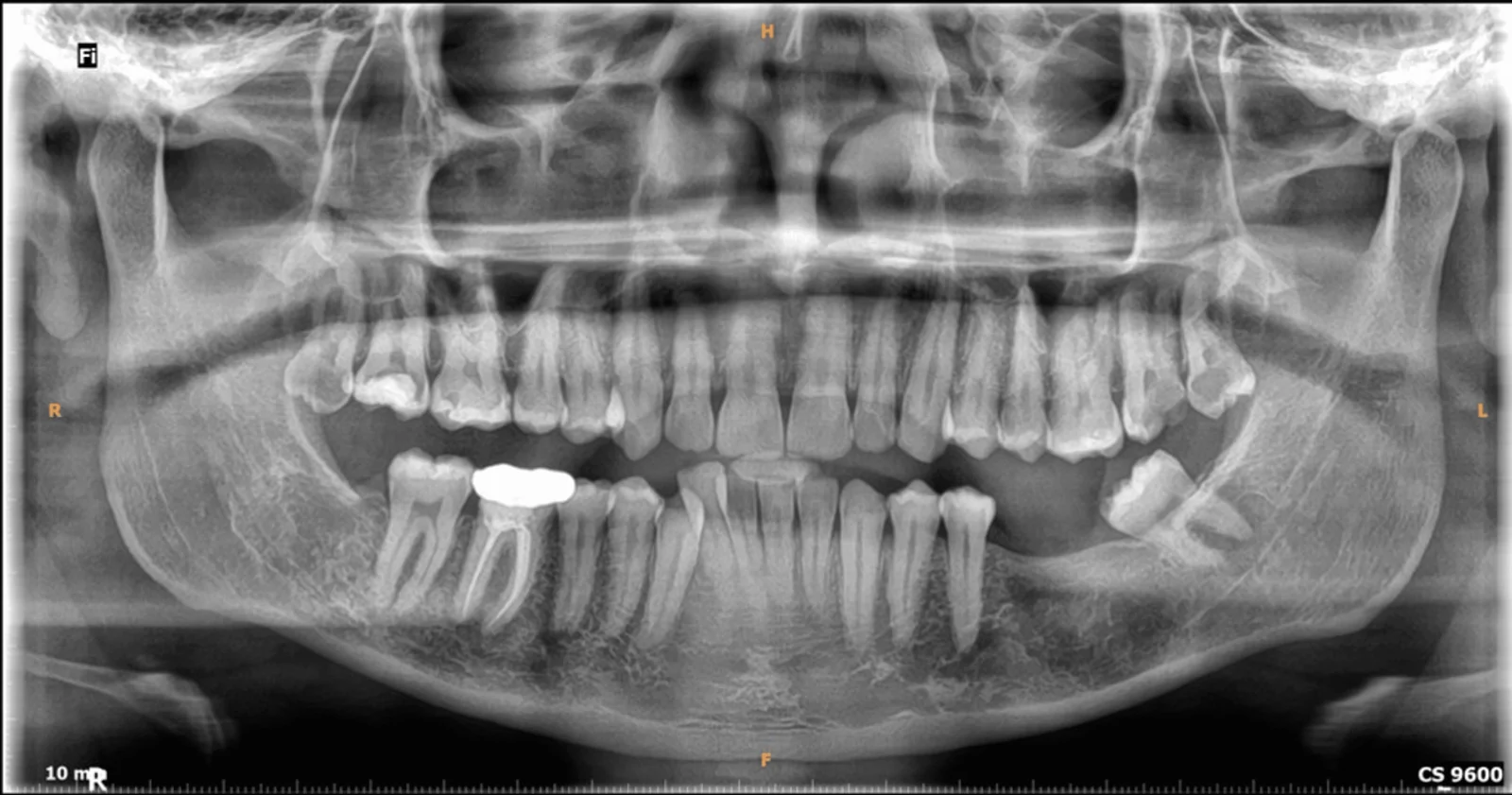
OPG used for grading the visualization of the inferior alveolar canal and mental foramen OPG: orthopantomogram

**Figure 2:**
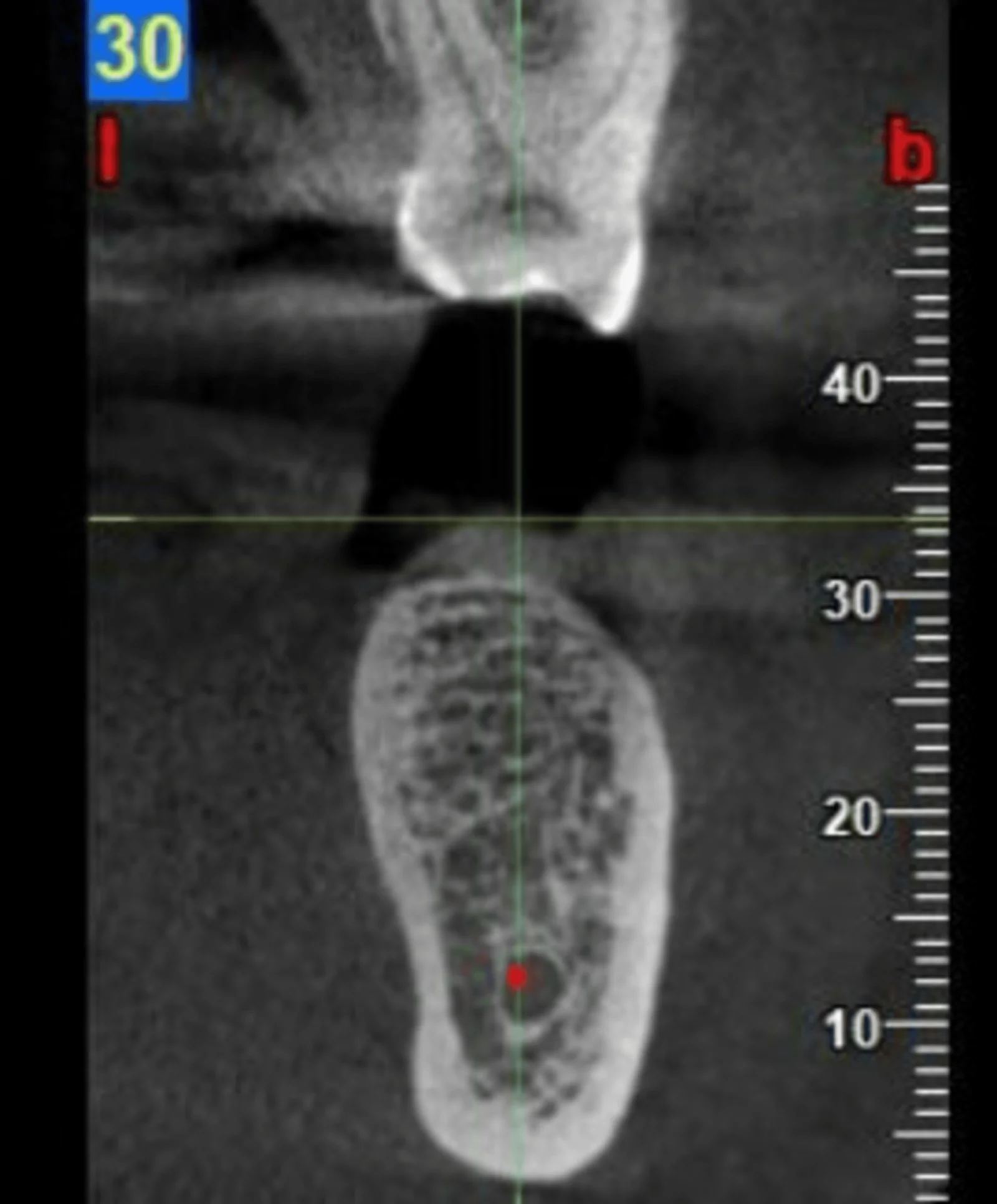
Coronal section of CBCT used for grading the visualization of the inferior alveolar canal CBCT: cone-beam computed tomography

Qualitative Assessment of Vital Structures at the Implant Site

For each intended implant site, visibility of the crest of the alveolar ridge and the superior border of the inferior alveolar canal was assessed on both OPG and CBCT images using the same four-point grading system. These structures define the vertical envelope of bone available for safe implant placement and the minimum distance to the neurovascular bundle.

Quantitative Measurements of Bone Dimensions

On OPGs, measurements were performed using a magnifying lens and Vernier calipers with a precision of 0.1 mm, with the films viewed on an illuminated view box. Two primary parameters were recorded (Figure 3): bone height, which is the distance from the crest of the alveolar ridge to the superior border of the mandibular canal at the planned implant site; and mesiodistal width, which is the length of the edentulous span available for implant placement.

**Figure 3:**
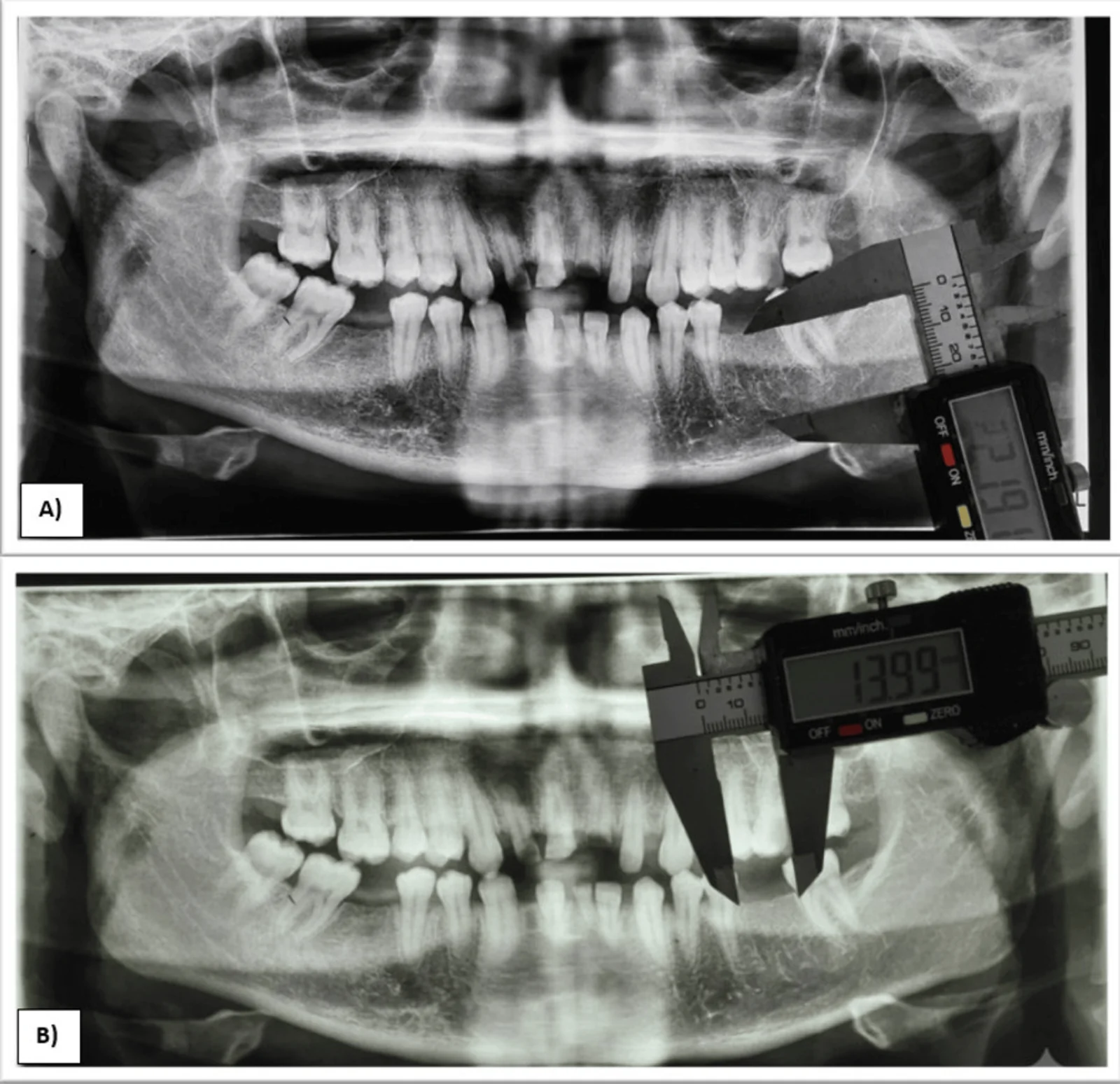
OPG assessment of the alveolar bone dimensions OPG: orthopantomogram (a) Assessment of the height of the bone from superior border of the inferior alveolar canal to the crest of alveolar ridge in OPG. (b) Assessment of the mesiodistal width of bone in OPG

On CBCT, analogous measurements were obtained using software tools (Figure 4): bone height, which was measured on cross‑sectional or coronal slices; mesiodistal width, which was measured on sagittal slices corresponding to the edentulous segment; and buccolingual width, which was measured on coronal slices; this dimension cannot be reliably obtained from panoramic radiography.

**Figure 4:**
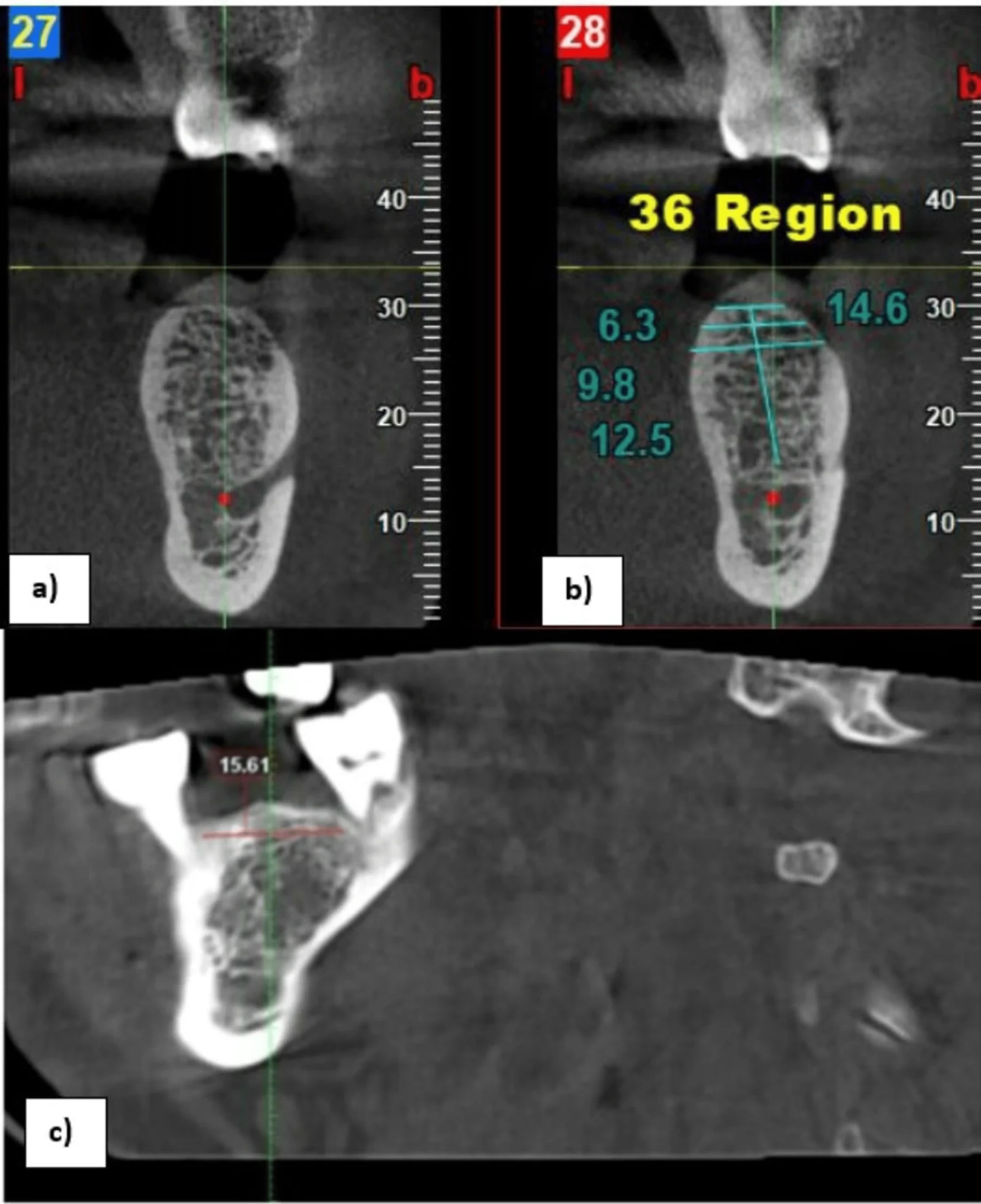
CBCT assessment of alveolar bone dimensions CBCT: cone-beam computed tomography (a) Assessment of the height of bone from the superior border of the inferior alveolar canal to the crest of the alveolar ridge in coronal section of the CBCT. (b) Assessment of the buccolingual width of the bone in coronal cut of CBCT. (c) Assessment of the mesiodistal width of the bone in sagittal cut of the CBCT

Bone Density Assessment

Bone density at the implant site was assessed exclusively on CBCT images using Hounsfield unit (HU) values derived from the voxel data. Based on HU ranges, sites were categorized according to the Misch classification [9-11]: D1, dense cortical bone (> 1250 HU); D2, thick, dense to porous cortical bone with coarse trabecular bone (850-1250 HU); D3, thin porous cortical bone with fine trabecular bone (350-850 HU); D4, fine trabecular bone (150-350 HU); and D5, immature, nonmineralized bone (<150 HU). OPGs do not permit reliable HU-based density measurements and were, therefore, not used for density assessment.

Statistical analysis

Data were entered into IBM SPSS Statistics for Windows, Version 16 (Released 2007; IBM Corp., Armonk, New York, United States) for analysis. Descriptive statistics were presented as mean ± standard deviation (SD) for continuous variables and as frequencies and percentages for categorical variables. As this was a paired, within-subject design with ordinal grading outcomes, between-modality comparisons for the four-point visibility grading scores were carried out using the Wilcoxon signed-rank test, with results reported as median (interquartile range (IQR)) alongside mean ± SD. Between-modality comparisons for continuous linear measurements (bone height, mesiodistal width) were carried out using the paired t-test. Buccolingual width and bone density were assessable only on CBCT and are reported descriptively, without inferential comparison to panoramic radiography, as these parameters cannot be measured on panoramic radiography.

## Results

Mandibular canal visibility at the mental foramen and distal sites

The distribution of grading scores for mandibular canal visibility in OPGs and CBCT in four places (at the mental foramen, 1 cm, 2 cm, and 3 cm posterior to the mental foramen) is shown in Table 1. CBCT visualized the mandibular canal better than OPG in all four regions. At the mental foramen, 0% excellent (Grade 4) visibility was observed in OPG, and 65.3% poor visibility (Grade 1) of the mandibular canal at the mental foramen in OPG and 61.5% in CBCT were observed. A high number of severe limitations (Grade 2) were observed at 3 cm from the mental foramen in OPG, and 61.5% excellent visibility (Grade 4) was seen at 3 cm from the mental foramen in CBCT. Also, 0% poor visualization (Grade 1) of the mandibular canal was observed in the CBCT at 2 cm and 3 cm from the mental foramen. CBCT showed significantly better visualization of the mandibular canal than OPG at all four locations tested: at the mental foramen (p = 0.0094), at 1 cm (p = 0.0013), at 2 cm (p = 0.0002), and at 3 cm (p = 0.0002) posterior to the mental foramen (Table 1). The difference between modalities increased with distance from the mental foramen.

**Table 1:** Distribution of grading score and mean ± SD for comparison of visualization of the mandibular canal at the mental foramen between OPG and CBCT OPG: orthopantomogram; CBCT: cone-beam computed tomography; IQR: interquartile range (*p < 0.05, significant compared with panoramic radiograph using CBCT)

Location	Modality	Grade 1 n (%)	Grade 2 n (%)	Grade 3 n (%)	Grade 4 n (%)	Mean ± SD	Median (IQR)	Z statistic	p-value
Mental foramen	OPG	17 (65.4)	6 (23.1)	3 (11.5)	0 (0.0)	1.46 ± 0.71	1.0 (1.0-2.0)		
	CBCT	16 (61.5)	2 (7.7)	3 (11.5)	5 (19.2)	1.88 ± 1.24	1.0 (1.0-3.0)	-2.60	0.0094
1 cm	OPG	12 (46.2)	5 (19.2)	7 (26.9)	2 (7.7)	1.96 ± 1.04	2.0 (1.0-3.0)		
	CBCT	7 (26.9)	9 (34.6)	2 (7.7)	8 (30.8)	2.42 ± 1.21	2.0 (1.25-4.0)	-3.21	0.0013
2 cm	OPG	7 (26.9)	6 (23.1)	5 (19.2)	8 (30.8)	2.54 ± 1.21	2.5 (1.25-4.0)		
	CBCT	0 (0.0)	7 (26.9)	7 (26.9)	12 (46.2)	3.19 ± 0.85	3.0 (2.25-4.0)	-3.69	0.0002
3 cm	OPG	0 (0.0)	10 (38.5)	7 (26.9)	9 (34.6)	2.96 ± 0.87	3.0 (2.0-4.0)		
	CBCT	0 (0.0)	3 (11.5)	7 (26.9)	16 (61.5)	3.50 ± 0.71	4.0 (3.0-4.0)	-3.74	0.0002

The mean and standard deviation of grading of visibility of vital structures (crest of the alveolar ridge and the superior border of the inferior alveolar canal) in OPGs and CBCT are shown in Table 2. CBCT shows 0% poor visibility (Grade 1) of both vital structures, whereas 53.8% of images show excellent visibility of the crest of the alveolar ridge and 50.0% show Grade 3 for visualization of the superior border of the inferior alveolar canal. CBCT demonstrated significantly clearer visualization of both the superior border of the inferior alveolar canal (p < 0.0001) and the crest of the alveolar ridge (p < 0.0001) compared with OPG (Table 2).

**Table 2:** Distribution of grading score and mean ±SD for comparison of vital structures at the implant site in mandible between OPG and CBCT OPG: orthopantomogram; CBCT: cone-beam computed tomography; IQR: interquartile range (*p < 0.05 significant compared to panoramic radiograph with CBCT)

Structure	Modality	Grade 1 n (%)	Grade 2 n (%)	Grade 3 n (%)	Grade 4 n (%)	Mean ± SD	Median (IQR)	Z statistic	p-value
Superior border IAN	OPG	7 (26.9)	13 (50.0)	6 (23.1)	0 (0.0)	1.96 ± 0.72	2.0 (1.25-2.0)		
	CBCT	0 (0.0)	7 (26.9)	13 (50.0)	6 (23.1)	2.96 ± 0.72	3.0 (2.25-3.0)	-4.74	<0.0001
Crest of alveolar ridge	OPG	0 (0.0)	6 (23.1)	19 (73.1)	1 (3.8)	2.81 ± 0.49	3.0 (3.0-3.0)		
	CBCT	0 (0.0)	0 (0.0)	12 (46.2)	14 (53.8)	3.54 ± 0.51	4.0 (3.0-4.0)	-4.36	<0.0001

Additionally, OPG and CBCT were compared to assess the quantity and quality of available bone by measuring the height of the bone from the crest of the alveolar ridge to the superior border of the inferior alveolar canal, mesiodistal width, buccolingual width, and density of bone in HU at the implant site. The mean and standard deviation of these parameters are shown in Table 3.

**Table 3:** Statistics of comparison of quality and quantity of available bone at the implant site in mandible between OPG and CBCT OPG: orthopantomogram; CBCT: cone-beam computed tomography; IQR: interquartile range; SD: standard deviation; LoA: limit of agreement; HU: Hounsfield unit; MD: mesiodistal; BL: buccolingual Bias represents the mean paired difference between CBCT and OPG measurements (CBCT-OPG), calculated using the Bland-Altman method. The 95% limits of agreement (mean bias ± 1.96 × SD of the paired differences) indicate the range within which most differences between the two modalities are expected to fall for an individual patient

Parameter	OPG	CBCT	Bias (95% LoA)	t-stat (df)	p-value
Height of bone (mm)	14.30 ± 2.35	14.45 ± 2.24	0.15 (-1.58, 1.89)	t(25) = 0.888	0.383
MD width (mm)	12.50 ± 2.32	12.76 ± 2.33	0.22 (-0.94, 1.37)	t(25) = 1.860	0.075
BL width (mm)	N/A	9.42 ± 1.76	N/A	N/A	N/A
Density (HU)	N/A	1193.23 ± 90.31	N/A	N/A	N/A

Height and mesiodistal width showed no significant difference between panoramic radiography and CBCT (height: paired t(25) = 0.888; p = 0.383; mesiodistal width: paired t(25) = 1.860; p = 0.075). Buccolingual width and bone density in HU could only be assessed on CBCT, as these parameters are not measurable on panoramic radiography; therefore, no direct statistical comparison between modalities was performed for these two parameters.

## Discussion

This study confirms that CBCT offers superior visualization of the mandibular canal in the posterior mandible compared with OPG, particularly at sites located 2-3 cm away from the mental foramen. Poor canal visualization on panoramic images in anterior regions is likely due to anatomical factors such as thinning or absence of cortical canal roofs and superimposition of radiolucent structures, combined with the inherent limitations of 2D projection imaging.

The findings of the present study are consistent with previous investigations showing that CT or CBCT delineates the mandibular canal more reliably than panoramic or periapical radiography [12-14]. The current study adds clinical evidence using a routinely available CBCT unit and standardized grading criteria in direct comparison with conventional OPG.

Clinical implications for presurgical implant planning

Clinically, accurate localization of the mandibular canal and determination of the vertical distance from the alveolar crest are critical to avoid iatrogenic damage to the inferior alveolar nerve during implant placement. In sites where OPG provides unclear canal contours or suggests limited vertical bone height above the canal, CBCT offers important supplementary information by visualizing the canal course and its buccolingual position relative to the crest. Because CBCT can depict buccolingual dimensions and cortical thickness, it allows clinicians to evaluate ridge morphology comprehensively, select appropriate implant diameter and length, and anticipate the need for bone augmentation procedures. Additionally, HU-based density estimation provides an objective measure of bone quality that can be correlated with insertion torque and implant stability, as reported in external literature, thereby informing surgical strategies such as drilling protocols and loading schedules.

Key findings

CBCT visualized the mandibular canal significantly more reliably than OPG at all four evaluated locations, with the magnitude of difference increasing distally. At the mental foramen, OPG showed predominantly poor visibility (grade 1 in 65.4% of sites; median, 1.0; IQR: 1.0-2.0), while CBCT achieved a higher median score (median: 1.0; IQR: 1.0-3.0; p = 0.0094). At 1 cm posterior, OPG's median score was 2.0 (IQR: 1.0-3.0) compared with CBCT's median of 2.0 (IQR: 1.25-4.0; p = 0.0013). The largest differences were seen more distally: at 2 cm, OPG had a median of 2.5 (IQR: 1.25-4.0) versus CBCT's 3.0 (IQR: 2.25-4.0; p = 0.0002), and at 3 cm, OPG's median was 3.0 (IQR: 2.0-4.0) versus CBCT's 4.0 (IQR: 3.0-4.0; p = 0.0002).

CBCT also showed significantly better visualization of both vital structures at the implant site. For the crest of the alveolar ridge, OPG showed a median score of 3.0 (IQR: 3.0-3.0) compared with CBCT's 4.0 (IQR: 3.0-4.0; p < 0.0001). For the superior border of the inferior alveolar canal, OPG's median score was 2.0 (IQR: 1.25-2.0) versus CBCT's 3.0 (IQR: 2.25-3.0; p < 0.0001).

Bone Dimensions and Density

Quantitatively, the mean vertical bone height from the alveolar crest to the superior canal border was 14.30 ± 2.35 mm on panoramic radiographs and 14.45 ± 2.24 mm on CBCT (bias: 0.15 mm; 95% LoA: -1.58 to 1.89; p = 0.383), indicating very close agreement between modalities. Similarly, the mean mesiodistal width at the implant site was 12.50 ± 2.32 mm on panoramic radiography and 12.76 ± 2.33 mm on CBCT (bias: 0.22 mm; 95% LoA: -0.94 to 1.37; p = 0.075). These findings support the use of panoramic radiographs for preliminary assessment of height and mesiodistal span in straightforward cases.

By contrast, buccolingual width and bone density were assessable only on CBCT. OPG, by design, recorded 0.00 ± 0.00 mm for buccolingual width and 0.00 ± 0.00 for density in HU, whereas CBCT yielded a mean buccolingual width of 9.42 ± 1.76 mm, a parameter not assessable on panoramic radiography, and a mean bone density of 1193.23 ± 90.31 HU (range: 1010-1345 HU), likewise not assessable on panoramic radiography. The raw data sheet further shows individual buccolingual widths ranging approximately from 5.0 to 13.3 mm and HU‑based bone quality mostly in the D1-D2 range (e.g., 1010-1345 HU), indicating generally favorable density in this sample. These numerical results emphasize that OPG can approximate vertical height and nasal distal width but cannot inform decisions about implant diameter, need for augmentation, or expected primary stability, all of which depend heavily on buccolingual quality.

The quantitative superiority of CT for canal localization in this study agrees with earlier work by Klinge et al., Lindh et al., and Sonick et al. [12-14], who reported more accurate canal positioning and less dimensional distortion with CT than with panoramic radiography, especially in posterior mandibular sites. Angelopoulos et al. [15] similarly found that CBCT-reformatted panoramic images outperform digital OPGs for mandibular canal identification, while Oliveira-Santos et al. [16] observed better canal visibility in digital regions on CBCT, consistent with our significantly higher grading scores at 2 cm and 3 cm posterior to the mental foramen. Unlike several of these earlier studies, which relied on macroscopic sectioning, hypocycloidal tomography, or research-grade CT/CBCT protocols not typically available in routine clinical practice, the present study used a standard clinically deployed CBCT unit and evaluated visibility at four standardized distances from the mental foramen alongside quantitative HU-based density classification in the same patient cohort. This combination of standardized ordinal grading, linear measurement, and density classification within a single paired comparison has not been consistently reported together in prior panoramic-versus-CBCT studies, most of which addressed only one or two of these dimensions in isolation.

The close agreement in height and mesiodistal width between modalities (differences of 0.15 mm and 0.22 mm, respectively) mirrors ex vivo and clinical studies showing that panoramic or ridge mapping measurements differ from CT/CBCT by less than 1 mm in many cases, with limited impact on routine planning [17-19]. However, as in studies by Shiratori et al. [20], our data confirm that CBCT yielded a mean buccolingual width of 9.42 ± 1.76 mm, a parameter not assessable on panoramic radiography, and a mean bone density of 1193.23 ± 90.31 HU (range: 1010-1345 HU), likewise not assessable on panoramic radiography.

Finally, the HU values and Mish classification in our data set (numerous sites in D1-D2 with HU often above 1000) align with previous reports that CBCT/CT-derived density correlates with insertion torque and implant stability, offering objective support for surgical decision-making [21-24]. OPG, by contrast, cannot provide such quantitative density data.

Clinical implications

Numerically, the study shows that CBCT transforms canal visibility from predominantly poor (65.3% grade 1 at the mental foramen on OPG) to substantially better visualization more posteriorly (61.5% grade 4 at 3 cm on CBCT), while also adding a mean buccolingual width of about 9 mm and HU‑based bone quality information that OPG entirely lacks. Therefore, for posterior mandibular implants near the canal or mental foramen, or when buccolingual width and bone density are critical, CBCT should be preferred. OPG can still be considered adequate when vertical bone height is clearly sufficient, and the canal is well‑delineated, but its numerical limitations in width and density assessment must be recognized

Limitations

The limitations of this study include its retrospective design, convenience sampling, and the subjective nature of visibility grading without formal interobserver reliability analysis. Ordinal visibility grading data were analyzed using the Wilcoxon signed-rank test, appropriate for paired ordinal outcomes; continuous linear measurements were analyzed using the paired t-test. HU values from CBCT may vary with machine calibration and exposure parameters, and the use of a single institution and specific CBCT/panoramic systems may limit generalizability. Future prospective, multicenter studies with standardized CBCT calibration and outcome measures such as neurosensory status and implant survival would help clarify the impact of imaging modality choice on patient safety and long‑term success.

## Conclusions

In conclusion, the study findings suggest that CBCT images of the mandibular canal and buccolingual width of the implant site are much better than traditional panoramic radiography. CBCT provided significantly better visualization of the mandibular canal at all evaluated distances from the mental foramen, with the greatest advantage seen more posteriorly, and should be considered whenever panoramic radiography does not clearly delineate the canal or vital structures at the implant site. CBCT provided consistently clearer visualization of the mandibular canal and crest of the alveolar ridge in the posterior mandible than panoramic radiography, particularly in sites distal to the mental foramen. It also uniquely allowed assessment of buccolingual ridge width and bone density, which are crucial for selecting implant dimensions, planning augmentation, and anticipating primary stability. Panoramic radiography, while limited to two‑dimensional information, still offered reasonably accurate estimates of vertical bone height and mesiodistal span and may be adequate for preliminary planning in uncomplicated cases with favorable anatomy and a clear safety margin to the canal. Overall, the study supports selective use of CBCT for posterior mandibular implant planning when canal visualization on panoramic radiography is suboptimal or when full three‑dimensional evaluation of the ridge and bone quality is required.
